# Screening and Prevention for High-Grade Serous Carcinoma of the Ovary Based on Carcinogenesis—Fallopian Tube- and Ovarian-Derived Tumors and Incessant Retrograde Bleeding

**DOI:** 10.3390/diagnostics10020120

**Published:** 2020-02-22

**Authors:** Isao Otsuka, Takuto Matsuura

**Affiliations:** Department of Obstetrics and Gynecology, Kameda Medical Center, Kamogawa 296-8602, Japan; matsuura.takuto@kameda.jp

**Keywords:** ovarian cancer, high-grade serous carcinoma, fallopian tube, screening, prevention, incessant retrograde bleeding

## Abstract

High-grade serous carcinoma (HGSC) is the most common and lethal subtype of ovarian carcinoma. Many HGSCs are now believed to originate in the fallopian tube epithelium; ovarian surface epithelium is another possible origin. Thus, current screening methods, i.e., ultrasonography and serum CA-125 measurements, have a limitation in their early detection. Recently, circulating biomarkers, such as tumor DNA, autoantibody, and microRNA, have been investigated to detect HGSCs. As cancer cells in the fallopian tube flow into the endometrial cavity, the detection of exfoliated cells, tumor DNA, and proteome from samples obtained from the endometrial cavity or the cervix may be useful. The risk of ovarian serous carcinoma is affected by the use of oral contraceptive and menopausal hormone therapy (MHT). MHT regimens causing endometrial bleeding increase serous carcinoma risk, hence, incessant retrograde bleeding from the endometrial cavity into the Douglas pouch appears to play an important role in high-grade serous carcinogenesis. In this review, we provide an overview of current and novel screening methods and prevention approaches for ovarian and fallopian tube HGSC.

## 1. Introduction

Ovarian cancer is the most lethal gynecological malignancy since most cases are diagnosed at an advanced stage when the metastases are extensive. In an effort to detect ovarian cancer at an early stage when the disease has a more favorable prognosis, ovarian cancer screening has been performed for more than three decades [1,2]. However, unfortunately, current methods for ovarian cancer screening, i.e., transvaginal ultrasonography (TV-US) and serum cancer antigen (CA)-125 measurements, did not reduce the mortality rate [3,4,5]. Hence, routine screening of the general population for ovarian cancer is not recommended at present [6].

Recently our understanding of the origins and pathogenesis of ovarian cancer has substantially progressed, and novel methods for early detection of ovarian cancer have been developed. Although the etiology of ovarian cancer remains unclear, some novel hypotheses have been proposed to explain epidemiological risk factors. In this review, we discuss current and novel screening methods for ovarian cancer, in particular, high-grade serous carcinoma (HGSC), based on its carcinogenesis. Additionally, we propose a hypothesis of its etiology, incessant retrograde bleeding, and address primary prevention for HGSC.

## 2. Ovarian Cancer Screening: Current Methods and Limitations

In the general population, ovarian cancer screening using TV-US and serum CA-125 measurements has detected many early-stage cancers. However, three randomized trials using these methods did not detect the disease at an early stage or achieve a mortality reduction in asymptomatic postmenopausal women [3,4,7]. Kobayashi et al. investigated the efficacy of screening with sequential pelvic ultrasound and serum CA-125 test in 82,467 postmenopausal women in Japan and reported that the proportion of stage I ovarian cancer was higher in the screened group than in the control group (63% vs. 38%), but the difference was not statistically significant (*p* = 0.229) [7]. Buys et al. investigated 78,216 women aged 55 to 74 years in the United States and concluded that among women in the general population, simultaneous screening with CA-125 and TV-US did not reduce ovarian cancer mortality compared with usual care. They also reported that a false-positive screening test result led to complications with unnecessary surgery [3]. Jacobs et al. investigated 202,638 postmenopausal women aged 50 to 74 years in the United Kingdom and reported that the mortality reduction was not significant, whereas a significant mortality reduction with multimodal screening was observed when prevalent cases (diagnosed at the first screen visit) were excluded [4].

The prevalence of ovarian cancer in the general population is relatively low (1:2500) [8], and higher prevalence was observed in women considered to be at high risk. High-risk women have cancer predisposing gene mutations (lifetime risk, 10–40%), i.e., those with *BRCA1/BRCA2* mutations or those with Lynch syndrome. Women at low-risk (lifetime risk, 1–2%) have no family history of ovarian cancer or a single first-degree relative with ovarian cancer, and women at moderate-risk (lifetime risk, 3–10%) have a more significant history but no mutations in *BRCA* genes [9]. However, even in women with a *BRCA1/2* mutation, who are actually at high risk of developing ovarian cancer among women with family history [10], ovarian cancer screening using TV-US and CA-125 measurements did not detect ovarian cancer at an early stage [11,12].

Although the sensitivity of ovarian cancer screening using TV-US in a study was 87% [13], which outperformed screening mammography [14], the limitations of screening using current methods can be partly explained by its limitations in detecting HGSC, which is the most common and lethal subtype of ovarian cancer.

## 3. Pathogenesis of High-Grade Serous Carcinoma: Fallopian Tube- and Ovarian-Derived Tumors 

### 3.1. Ovarian Carcinoma, a Group of Distinct Diseases

Epithelial ovarian cancer is not a single disease, but a heterogeneous group of neoplasms that are classified into five main cell types: high-grade serous, low-grade serous, endometrioid, clear cell, and mucinous carcinomas. These five subtypes are essentially distinct tumors because they not only behave differently but also develop differently [15,16,17]. Additionally, ovarian cancers can be divided into type I and II tumors [16]. Type I tumors include low-grade serous, endometrioid, clear cell, and mucinous carcinomas. Type II tumors include high-grade serous and endometrioid carcinomas. Type I tumors are suggested to develop from benign extraovarian lesions that implant on the ovary and that can subsequently undergo malignant transformation [16,17]. Thus, they are thought to arise from precursor lesions that develop in the ovary. Endometrioid and clear cell carcinomas appear to develop in ovarian endometriotic cysts and low-grade serous carcinomas appear to develop from borderline tumors [16,17]. Mucinous carcinoma is a rare subtype and its carcinogenesis is still unknown. Most type I tumors can be detected early by TV-US with or without CA-125 measurements [18]. In contrast, many type II carcinomas develop from intraepithelial carcinomas in the fallopian tube [16,17,19].

### 3.2. High-Grade Serous Carcinogenesis

Recent studies, in particular, studies on risk-reducing salpingo-oophorectomy (RRSO) performed in women with a *BRCA* mutation, have revealed that many HGSCs, i.e., the most common type II tumor, arise in the fallopian tube, particularly in the fimbriae [16,19]. A large majority of sporadic cases of HGSCs also arise in the fallopian tube [20]. *TP53* mutation is observed in almost all HGSC cells [21,22] and appears to be a driver mutation in the pathogenesis of HGSC [22]. In animal models, deletions of *Brca*, *Tp53*, and *Pten* lead to the development of HGSCs arising from fallopian tube secretory cells [23], and inactivation of several tumor suppressor genes, including *Brca* and *Trp53*, results in serous tubal intraepithelial carcinomas (STICs) [24].

In the fallopian tube surface epithelium, intraepithelial lesions associated with HGSC, i.e., p53 signature, serous tubal intraepithelial lesion (STIL), and STIC can be identified. p53 signature is characterized by a linear, strongly immunopositive segment of tubal cells spanning at least 12 consecutive secretory cell nuclei, and no cytological atypia and very low proliferative index. STILs are intermediate lesions between p53 signature and STIC, representing no cytological atypia, low/moderate proliferative index, but p53 accumulation. STICs are composed of secretory cells showing significant atypia, architectural alterations, high proliferative index, and strong p53 immunostaining [25].

p53 signature may not necessarily be associated with the development of HGSC. p53 signature was observed in 50% of women without a *BRCA* mutation who underwent a salpingectomy for benign diseases [26]. In contrast, STIC may be a putative precursor of HGSC [27]. STIC was observed only in 0.1–0.8% of low-risk women, such as women without a history of or known risk factors (*BRCA* mutation) for HGSC [28,29]. Another study reported that the incidence of STIC in women without *BRCA* mutation was much higher, 3%; in that study 8% of women with *BRCA* mutation had a STIC [30]. STIC has malignant cellular features, such as enlarged nuclei and prominent nucleoli, which are also characteristic histopathological features of HGSC. In a population-based study, although HGSC incidence rates were relatively stable, those of fallopian tube carcinoma in situ (an imperfect surrogate of STIC) increased recently, which appears to reflect increased detection with meticulous pathology processing protocols [31]. The existence of occult tubal carcinoma in situ may not affect long-term survival, as five-year cause-specific survival was 98%.

Ovarian surface epithelium (OSE) is also a cell of origin for HGSC. Many ovarian HGSCs, particularly advanced-stage disease, do not have STICs. The reported coexistence between STICs and HGSCs ranged from 11% to 61% (mean 31%) [32]. A genomic study reveals that a proportion of STICs represents intraepithelial metastases to the fallopian tube rather than the origin of HGSC [33]. In a mouse model, ovaries harboring a *p53* mutation develop metastatic HGSCs [34], and transcriptome data from OSE, fallopian tube epithelium (FTE), and HGSC samples reveal that HGSC has two subtypes originated from either FTE or OSE [35]. Interestingly, OSE-derived HGSC may have a worse prognosis and long latent period compared to FTE-derived HGSC [36,37].

In *BRCA* mutation carriers that underwent RRSO, the majority of microscopic cancers were observed in the fallopian tubes. In addition, microscopic cancers were observed only in the ovary or peritoneal washings in some women [38]. These observations may be explained by the phenomenon called “early precursor escape” [39]. Early serous proliferations, i.e., p53 signature and STILs, which lack cellular atypia but contain *TP53* mutations, can be shed from the fallopian tube and eventually undergo malignant transformation on the ovarian or peritoneal surface, as well as STICs.

## 4. Screening Methods for High-Grade Serous Carcinoma

The main goal of screening for a particular cancer is to reduce the mortality rate from that cancer among the persons screened. Hence, the detection of preinvasive lesions, or at least small curable lesions, is required for cancer screening.

### 4.1. Conventional Screening Methods

For early detection of ovarian cancer before the development of overt symptoms, imaging studies, such as ultrasonography, are useful (Table 1). Imaging studies can detect a lesion approximately >1 cm in diameter; hence, they may not be effective in HGSC screening, because cancer cells can exfoliate from the original lesion before it grows to a mass detectable by imaging studies. However, in asymptomatic women 50 years of age or older or women 25 years of age or older with a family history of ovarian cancer, women with type II ovarian tumors (the vast majority were HGSCs) that were detected by screening using ultrasonography had a significantly longer disease-specific survival than women with clinically detected type II tumors [13]. Thus, a certain number of ovarian HGSCs develop an ovarian tumor that can be detected with ultrasonography before they develop extensive metastases.

Serum CA-125 is the most sensitive tumor marker for ovarian cancer, whereas many serum biomarkers have been developed [40]. CA-125 is useful for the detection of a low-volume disease; however, in a mathematical model, ovarian cancer can reach a volume of π/6(25 mm)^3^ , corresponding to a spherical diameter of about 25 mm, before becoming detectable by current clinical blood assays [41].

### 4.2. Novel Screening Methods Based on Carcinogenesis

Many novel approaches have been investigated to overcome the limitations inherent to current screening methods, i.e., TV-US and CA-125 measurements. Blood testing may be beneficial to detect a smaller lesion. As a tumor grows, tumor cells invade into capillaries, and then tumor DNA and proteins are released from apoptotic and necrotic tumor cells into the bloodstream. Thus, circulating tumor DNA (ctDNA) and proteins can be detected in the blood, as well as autoantibodies to tumor DNA [42,43,44,45,46]. However, these biomarkers appear to be detected only after an invasive tumor becomes a certain size, as a load of circulating tumor DNA correlates with tumor staging [47].

Recent progress in cancer detection methods may allow the detection of precursor lesions of HGSC (Table 1). At a stage of an intraepithelial tumor, HGSC may be detected from materials other than blood. As carcinoma cells shed from the distal fallopian tube can flow into the endometrial cavity, endometrial cytological testing can detect these cells [48,49]. For endometrial cytological evaluation, samples are obtained from the endometrial cavity using a disposable plastic brush (endometrial sampler). The samples obtained are usually smeared directly on slides for fixation and staining. Cervicovaginal cytology can also detect carcinoma cells. We performed a review of the literature regarding ovarian and fallopian tube cancer detected by the endometrial and cervicovaginal cytological testing. References for this review were identified through searches of PubMed (for papers published in English) and Igaku Chuo Zasshi (Medical Central Journal; for papers published in Japanese) with the search terms ‘ovarian cancer’, ‘fallopian tube cancer’, ‘cervical smear (cytology)’, and ‘endometrial smear (cytology)’ from 1985 until December, 2014. Articles were also identified through searches of the authors’ own files. We only included cases in which there were no abnormalities on transvaginal ultrasonography, but fallopian tube or ovarian carcinomas were detected by examining cytological samples. Cases with cytological findings positive for malignant cells were included, but cases with atypical but not malignant cells were excluded. Additionally, patients in whom a presurgical serum CA-125 measurement was performed were included. The review revealed that these cytological tests can detect ovarian and fallopian tube carcinomas without abnormalities on imaging studies [49,50,51,52,53,54,55,56,57,58,59,60,61,62,63,64,65,66,67,68] (Table 2). Of note, in 17 of 23 cases (74%) serum CA-125 levels were not elevated.

Tumor DNA and tumor proteome can be detected from samples obtained from the endometrial cavity or cervix in women with endometrial and/or ovarian cancer [69,70,71,72,73]. Although in these studies patients with invasive carcinoma (stages I-IV) were investigated, these biomarkers can theoretically detect precursor lesions such as STIC. Falloposcopy may be effective in collecting atypical cells of the fimbria [74]. Biomarkers in the urine and exhaled breath samples may be useful in detecting preclinical cancer [75,76].

Circulating microRNAs appear to be effective in the early detection of ovarian cancer [77,78]. MicroRNA (miRNA) is a small (20–25 nucleotides), non-coding RNA that regulates gene expression post-transcriptionally, and stable in the blood. A panel using eight miRNAs effectively detected invasive ovarian cancer including early-stage disease [77]. Additionally, the model could distinguish ovarian cancer patients from those with benign tumors.

Light-induced endogenous fluorescence can identify preinvasive lesions via falloposcopy [79]. An implantable optical sensor, which is composed of an antibody-functionalized carbon nanotube complex and placed proximal to disease sites, can detect HE4 in the patient biofluids [80].

For a screening test to be effective, it should be sensitive and specific, as well as cost-effective. In particular, a high positive predictive value is required to avoid unnecessary surgery which may cause morbidity. Among many novel screening methods that are now being investigated, only cytological testing appears to be available at present. This testing appears to have a high positive predictive value because STIC cells have severe nuclear atypia. Additionally, this testing method is inexpensive, well tolerated by patients, and easily performed by gynecologists in clinical settings [81]. However, limitations of endometrial cytological testing include low sensitivity, which was found to be 43% or less for HGSC in our previous study [49]. In women with cervical stenosis, which is observed more often in elderly women, this testing cannot be performed. Additionally, whether endometrial cytological testing can detect carcinomas early enough to improve outcome has yet to be determined. Based on our previous study [49] and a review of the literature, we have been performing an ovarian cancer screening program consisting of TV-US, CA-125, and endometrial cytological tests in high-risk women who do not want to undergo RRSO. Other novel methods, such as molecular and endoscopic methods, are necessary to be clinically validated in a prospective cohort.

## 5. Incessant Retrograde Bleeding—An Etiologic Factor in High-Grade Serous Carcinogenesis

Since current screening methods are not effective in reducing ovarian cancer mortality, primary prevention of ovarian cancers should be considered. To develop effective prevention methods, identification of risk factors is necessary.

### 5.1. Risk Factors of Ovarian Carcinoma and Carcinogenesis Hypotheses

Epidemiological studies have shown that protective factors of ovarian carcinoma include oral contraceptive (OC) use, parity, and breastfeeding [82,83]. These factors are associated with ovulation suppression; hence, the incessant ovulation hypothesis was postulated [84]. Constant damage and repair of the OSE lead to an increased risk of malignant transformation [85]. Ovulation-related inflammation may play a role in high-grade serous carcinogenesis: follicular fluid exposure causes up-regulation of inflammatory and DNA repair pathways and double-stranded DNA breaks are induced [86]. However, this hypothesis does not explain why tubal ligation reduces ovarian cancer risk [87], after which the incessant menstruation hypothesis has been postulated [88]. Retrograde menstruation from the endometrial cavity into the Douglas pouch and subsequent iron-induced oxidative stress in bloody fluid are the causative mechanisms. Fimbriae floating in bloody peritoneal fluid are exposed to the action of catalytic iron and the genotoxic effect of reactive oxygen species [88]. Transferrin-containing fluid, such as retrograde menstrual blood, may induce DNA double-strand breaks that potentially lead to DNA damage/genome instability [89]. Whereas incessant menstruation hypothesis explains ovarian cancer risk well in the premenopausal period, another risk factor is associated with ovarian carcinogenesis in menopausal women.

### 5.2. Incessant Retrograde Bleeding Hypothesis

Menopausal hormone therapy (MHT), also called hormone replacement therapy, increases ovarian cancer risk [90,91,92]. Considering this effect, incessant retrograde bleeding, which is an expansion of the concept of incessant menstruation, may explain more accurately the etiology of HGSC in menopausal women. In menopausal women experiencing vasomotor symptoms, estrogen therapy represents the most effective treatment [93], but progestin is also given to prevent endometrial cancer whereas breast cancer risk is increased with estrogen plus progestin [94]. MHT regimens are classified into three types: estrogen alone, estrogen with sequentially added progestin (sequential E + P), and continuous estrogen and progestin (continuous E + P). The use of estrogen alone causes irregular endometrial bleeding (breakthrough bleeding) and sequential E + P causes regular endometrial bleeding (withdrawal bleeding). In contrast, continuous E + P usually causes atypical bleeding at first but results in endometrial atrophy with bleeding cessation [95].

The risk of serous ovarian cancer differs by the regimen of MHT (Table 3). Its risk in women with intact uteri is increased with the use of estrogen alone and sequential E + P [90,92], but the use of continuous E + P does not change the risk [92]. Thus, regimens that cause endometrial bleeding, which is usually associated with retrograde bleeding into the Douglas pouch, are associated with increased risk of serous ovarian carcinoma. In the past, women with intact uteri also received estrogen alone, but currently, only hysterectomized women are treated with this regimen. The risk of fallopian tube carcinomas, almost all of which are thought to be HGSC, was increased with the use of sequential E + P but did not change with the use of continuous E + P [91]. Of note, estrogen alone did not change the risk of fallopian tube carcinoma in hysterectomized women [91]. In contrast, estrogen alone increased the risk of serous ovarian carcinoma in hysterectomized women, particularly for five or more years of use [92], although it does not cause retrograde bleeding. This discrepancy may be explained by the grade of serous carcinoma that develops after MHT use. Low-grade serous carcinoma develops from serous cystadenoma in the ovary, and ovarian serous cystadenoma is suggested to arise from epithelial inclusion glands that originate in the fallopian tube [96]. As estrogen stimulates proliferation of the fallopian tube epithelial cells [97], the majority of ovarian serous carcinoma that develops after long-term use of estrogen may be low-grade serous carcinoma, similar to type I endometrial carcinoma that develops after estrogen stimulation.

## 6. Prevention of High-Grade Serous Carcinoma

### 6.1. Surgical Prevention

Surgical risk reduction, particularly RRSO, plays an important role in the prevention of ovarian carcinoma in high-risk women. RRSO was associated with an 85% reduction in *BRCA1*-associated gynecological (ovarian, fallopian tube, or primary peritoneal) cancer risk [98]. In that study, protection against *BRCA2*-associated gynecological cancer was suggested, but the effect did not reach statistical significance. RRSO is recommended before age 40, but ideally by age 35, for women with a *BRCA1* mutation and before age 45 for women with a *BRCA2* mutation [99]. However, RRSO performed in premenopausal women causes endocrine symptoms and sexual symptoms associated with a decrease in estrogen levels, in addition to psychological distress. To avoid these adverse consequences of premature menopause, new preventive measures, such as bilateral salpingectomy with delayed oophorectomy, have been investigated [100]. However, ovarian preservation may place patients at risk for ovarian cancer which develops from OSE, a lesion formed by precursor escape [39] or from residual fimbrial tissue remaining of the ovarian surface [101]. For average-risk women after the completion of childbearing, opportunistic salpingectomy during benign gynecological surgery, which appears to be safe and may offer some protection from ovarian cancer, is now recommended by several professional societies [102].

### 6.2. Non-Surgical Prevention (Chemoprevention)

In high-risk women, i.e., women with a *BRCA* mutation, chemoprevention may be considered [9]. The cumulative risks for ovarian cancer by age 70 years were estimated to be 59% for *BRCA1* carriers and 17% for *BRCA2* carriers [103], and germline *BRCA1* and *BRCA2* mutations were exclusively associated with high-grade serous histology [104]. It appears that incessant retrograde bleeding plays a key role in the pathogenesis of HGSC; the reduction of this bleeding can reduce the HGSC risk. In women with a *BRCA* mutation, combined OC use reduces ovarian cancer risk [105,106]. Recently, extended and continuous regimens of combined OC can be utilized [107], and these regimens may be more effective than monthly OCs which are associated with monthly bleeding. Additionally, anti-inflammatory drug use during a bleeding period may reduce ovarian cancer risk [108]. Although OC use may increase breast cancer risk slightly, the overall cancer risk, including ovarian and endometrial cancer, may still be lower in OC users [109]. Of note, the use of progestogen-only products for hormonal contraception was not associated with ovarian cancer risk [110], whereas progesterone could eliminate p53-defective fallopian tube cells [111].

In menopausal women with intact uteri, continuous estrogen and progestin regimens should be given as MHT for <5 years [112]. In hysterectomized women with menopausal symptoms, estrogen-only should be administered for <7 years [112].

## 7. Concluding Remarks

Ovarian cancer screening needs to detect both early fallopian tube and ovarian lesions, as FTE and OSE are cells-of-origin for HGSC of the ovary [34,35,36,37]. Although ovarian cancer screening using TV-US and CA-125 is not recommended in low-risk women and high-risk women with *BRCA* mutations, for *BRCA* mutation carriers who have not yet undergone RRSO ovarian cancer screening may be considered at age 30–35 years [113]. In moderate-risk women, screening using these methods with or without a novel method may be effective [13]. Of patients with ovarian cancer, 14.5–24% carried germ-line mutations in cancer-associated genes [114,115,116], including *BRCA1/2* mutations that were observed in 13.3–15.3% [117,118,119]. Women with mutations in moderate penetrance genes, such as *RAD51C*, *RAD51D,* and *BRIP1* mutations, have a lifetime risk of 5.2–12% [9,120] and may benefit from ovarian cancer screening. Older age is associated with having a carcinoma [121], and 69% of new cases occur among women ages 55 and older [17]. Since most ovarian cancers develop after menopause and spontaneous menopause occurs at a mean age of 51–52 years [93], the threshold for increased risk is around age 50. Thus, postmenopausal women ≥50 years of age with a family history of ovarian cancer may receive ovarian cancer screening. Many moderate penetrance genes, as well as *BRCA1/2*, are associated with homologous recombination DNA repair defects and they are most commonly observed in HGSCs [122], hence, effective screening methods are needed to detect precursor lesions during a window period between the development of a STIC and initiation of invasive carcinoma, 6–7 years [123,124]. The fact that the incidence of ovarian carcinoma has been decreasing in recent years with the increase in OC use and the decrease in MHT use [125,126] may suggest the possibility of hormonal prevention of ovarian carcinoma.

HGSC is not a single disease but a group of neoplasms that include different transcriptional subtypes and different histopathological subtypes [21,127]. FTE-derived HGSC metastasizes rapidly and OSE-derived HGSC has longer latency, lower penetrance, and a worse prognosis [35,36]. To better select candidates for ovarian cancer screening, further studies are needed to identify subtype-specific risk factors and genotype-phenotype correlations and to explore tumor evolution.

## Figures and Tables

**Table 1:** Detection methods for high-grade serous carcinoma of the ovary and fallopian tube.

Theoretically Detectable Tumor Size	Detection Methods		Ref
Gross adnexal mass		TV-US	[2,13]
Small invasive carcinoma	Blood	CA-125 (+ TV-US)	[1,3,4,7]
		Tumor DNA	[42]
		DNA methylation	[43]
		Protein + Tumor DNA	[44]
		Glycoprotein	[45]
		Autoantibody	[46]
Intraepithelial neoplasia	Cervical mucus	Tumor DNA	[69,70,71]
	Endometrial sample	Cytology	[48,49]
	(lavage)	Tumor DNA	[72]
		Proteome	[73]
	Blood	Micro RNA	[77,78]
	Urine	Micro RNA	[75]
	Exhaled breath	Volatile gas	[76]
	Falloposcopy	Cytology	[74]
		Autofluorescence	[79]
	Biofluid (pelvis)	Optical nanosensor	[80]

**Table 2:** Cases of fallopian tube and ovarian cancer detected by endometrial/cervicovaginal cytology without abnormalities on imaging studies.

No.	Author (Year)	Age (Years)	Stage	CA-125 (U/mL)	CV/Em Cytology	Em Biopsy	Site	Histologic Type
Asymptomatic cases								
1	Otsuka (2013) [49]	58	0 ^A^	10	Pos/Pos	Neg	not specified	unknown
2	Narutomi (2001) [50]	72	Ic (TIC ^B^)	≤35	Pos/Pos	Neg	FT (Rt)	papillary
3	Safret (2004) [51]	36	Ic (TIC ^B^)	≤35	Pos/―	Neg	FT (Lt)	―
4	Doi (1991) [52]	52	Ic	11	Pos/Pos	Neg	FT (Rt)	papillary
5	Maeda (2010) [53]	57	Ic	≤35	―/Pos	Susp	FT (Bil)	serous
6	Yamakawa (1991) [54]	54	Ic	≤35	Pos/Pos	Neg	FT (Rt)	papillary
7	Iida (1989) [55]	40	Ic	48	Pos/Pos	Neg	FT (Lt)	papillary medullary
8	Konishi (2011) [56]	65	Ic	307	Susp/Pos	Neg	FT (Rt)	endometrioid
9	Warshal (1999) [57]	76	I ^C^	6	Pos/―	―	FT (Lt)	serous, G2
10	Kawanishi (2009) [58]	55	IIa	≤35	Neg/Pos	Neg	FT (Rt), Ov (Bil)	serous
11	Otsuka (2013) [49]	69	IIb	42	Susp/Pos	Neg	FT (Lt), Ov (Lt)	serous, G3
12	Ikarashi (1995) [59]	57	IIb (TIC ^B^)	<9	Pos/Pos	Neg	FT (Lt), Ov (Lt)	papillary
13	Otsuka (2013) [49]	55	IIb	47	Neg/Pos	Neg	FT (Rt), Ov (Lt)	serous, G2
Symptomatic cases								
14	Minato (1998) [60]	70	0 (TIC ^B^)	≤35	Neg/Pos	―	FT (Lt)	papillary
15	Fujimoto (1989) [61]	75	0 (TIC ^B^)	≤35	Pos/―	Neg	FT (Lt)	―
16	Imamura (2012) [62]	64	Ic	10.1	Pos/Pos	Neg	FT (Rt)	endometrioid
17	Iwamoto (2002) [63]	56	Ic	14.8	Neg/Pos	―	FT	―
18	Takeda (1991) [64]	69	Ic	19	Neg/Pos	Neg	Ov (Rt)	serous
19	Suzuki (1985) [65]	58	I	23	Neg/Pos	Neg	FT (Lt)	poorly-diff
20	Luzzatto (1996) [66]	57	I	115	Neg/Pos	―	FT (Lt)	―
21	Iwamoto (2002) [63]	52	IIIa (T2bN1)	9	Neg/Pos	―	FT, LNs	―
22	Miyao (2011) [67]	50s	IIIa (T3N1)	84.7	Neg/Pos	―	FT (Rt), Ov (Rt), Omentum, LNs	poorly-diff
23	Ohta (2009) [68]	64	IIIb	386	Neg/Pos	Neg	FT (Rt), Extrapelvis	endometrioid G3, clear cell

All symptomatic cases presented vaginal bleeding. ^A^ In this patient, the original tumor was unable to be found. ^B^ Fallopian tube lesion is intraepithelial carcinoma. ^C^ Fallopian tube carcinoma developed after vaginal hysterectomy. CV, cervicovaginal; Em, endometrial; ―, not reported; Neg, negative; Pos, positive; Susp, suspicious; FT, fallopian tube; Ov, ovary; Bil, bilateral; Rt, right; Lt, left; LN, lymph node; TIC, tubal intraepithelial carcinoma.

**Table 3:** Menopausal hormone therapy and risk of serous carcinoma.

	Estrogen Alone		Sequential Estrogen and Progestin	Continuous Estrogen and Progestin	Ref
	Hysterectomized Women	Women with Intact Uteri			
Ovarian cancer (serous)	↑	↑	↑	→	[90,92]
Fallopian tube cancer	→		↑	→	[91]

## References

[1] Jacobs I., Stabile I., Bridges J., Kemsley P., Reynolds C., Grudzinskas J., Oram D. (1988). Multimodal approach to screening for ovarian cancer. Lancet.

[2] Van Nagell J.R., Higgins R.V., Donaldson E.S., Gallion H.H., Powell D.E., Pavlik E.J., Woods C.H., Thompson E.A. (1990). Transvaginal sonography as a screening method for ovarian cancer. A report of the first 1000 cases screened. Cancer.

[3] Buys S.S., Partridge E., Black A., Johnson C.C., Lamerato L., Isaacs C., Reding D.J., Greenlee R.T., Yokochi L.A., Kessel B. (2011). Effect of screening on ovarian cancer mortality. The Prostate, Lung, Colorectal and Ovarian (PLCO) cancer screening randomized controlled trial. JAMA.

[4] Jacobs I.J., Menon U., Ryan A., Gentry-Maharaj A., Burnell M., Kalsi J.K., Amso N.N., Apostolidou A., Benjamin E., Cruickshank D. (2016). Ovarian cancer screening and mortality in the UK Collaborative Trial of Ovarian Cancer Screening (UKCTOCS): A randomised controlled trial. Lancet.

[5] Henderson J.T., Webber E.M., Sawaya G.F. (2018). Screening for ovarian cancer: An evidence review for the US Preventive Services Task Force. JAMA.

[6] US Preventive Services Task Force (2018). Screening for Ovarian Cancer US Preventive Services Task Force Recommendation Statement. JAMA.

[7] Kobayashi H., Yamada Y., Sado T., Sakata M., Yoshida S., Kawaguchi R., Kanayama S., Shigetomi H., Haruta S., Tsuji Y. (2008). A randomized study of screening for ovarian cancer: A multicenter study in Japan. Int. J. Gynecol. Cancer.

[8] Bast R.C., Matulonis U.A., Sood A.K., Ahmed A.A., Amobi A.E., Balkwill F.R., Wielgos-Bonvallet M., Bowtell D.D.L., Brenton J.D., Brugge J.S. (2019). Critical questions in ovarian cancer research and treatment: Report of an American Association for Cancer Research Special Conference. Cancer.

[9] Menon U., Karpinskyj C., Gentry-Maharaj A. (2018). Ovarian Cancer Prevention and Screening. Obstet. Gynecol..

[10] Maehle L., Apold J., Paulsen T., Hagen B., Lovslett K., Fiane B., van Ghelue M., Clark N., Moller P. (2008). High risk for ovarian cancer in a prospective series is restricted to BRCA1/2 mutation carriers. Clin. Cancer Res..

[11] Hermsen B.B.J., Olivier R.I., Verheijen R.H.M., van Beurden M., de Hullu J.A., Massuger L.F., Burger C.W., Brekelmans C.T., Mourits M.J., de Bock G.H. (2007). No efficacy of annual gynaecological screening in BRCA1/2 mutation carriers; an observational follow-up study. Br. J. Cancer.

[12] Van der Velde N.M., Mourits M.J.E., Arts H.J.G., de Vries J., Leegte B.K., Dijkhuis G., Oosterwijk J.C., de Bock G.H. (2009). Time to stop ovarian cancer screening in BRCA1/2 mutation carriers?. Int. J. Cancer.

[13] Van Nagell J.R., Burgess B.T., Miller R.W., Baldwin L., DeSimone C.P., Ueland E.R., Huang B., Chen Q., Kryscio R.J., Pavlik E.J. (2018). Survival of Women With Type I and II Epithelial Ovarian Cancer Detected by Ultrasound Screening. Obstet. Gynecol..

[14] Pavlik E.J., van Nagell J.R. (2013). Early detection of ovarian tumors using ultrasound. Womens Health.

[15] Prat J. (2012). New insights into ovarian cancer pathology. Ann. Oncol..

[16] Kurman R.J., Shih I.M. (2016). The dualistic model of ovarian carcinogenesis: Revisited, revised, and expanded. Am. J. Pathol..

[17] National Academies of Sciences, Engineering, and Medicine (2016). Ovarian Cancers: Evolving Paradigms in Research and Care.

[18] Nezhat F.R., Apostol R., Nezhart C., Pejovic T. (2015). New insights in the pathophysiology of ovarian cancer and implications for screening and prevention. Am. J. Obstet. Gynecol..

[19] Crum C.P., Drapkin R., Kindelberger D., Medeiros F., Miran A., Lee Y. (2007). Lessons from BRCA: The tubal fimbria emerges as an origin for pelvic serous cancer. Clin. Med. Res..

[20] Gilks C.B., Irving J., Köbel M., Lee C., Singh N., Wilkinson N., McCluggage W.G. (2015). Incidental Nonuterine High-grade Serous Carcinomas Arise in the Fallopian Tube in Most Cases Further Evidence for the Tubal Origin of High-grade Serous Carcinomas. Am. J. Surg. Pathol..

[21] Bell D., Berchuck A., Birrer M., Chien J., Cramer D.W., Dao F., Dhir R., DiSaia P., Gabra H., Glenn P. (2011). Integrated genomic analyses of ovarian carcinoma. Nature.

[22] Ahmed A.A., Etemadmoghadam D., Temple J., Lynch A.G., Riad M., Sharma R., Stewart C., Fereday S., Caldas C., Australian Ovarian Cancer Study Group (2010). Driver mutations in *TP53* are ubiquitous in high grade serous carcinoma of the ovary. J. Pathol..

[23] Perets R., Wyant G.A., Muto K.W., Bijron J.G., Poole B.B., Chin K.T., Chen J.Y.H., Ohman A.W., Stepule C.D., Kwak S. (2013). Transformation of the fallopian tube secretory epithelium leads to high-grade serous ovarian cancer in Brca; Tp53; Pten models. Cancer Cell.

[24] Zhai Y., Wu R., Kuick R., Sessine M.S., Schulman S., Green M., Fearon E.R., Cho K.R. (2017). High-grade serous carcinomas arise in the mouse oviduct via defects linked to the human disease. J. Pathol..

[25] Horn L.C., Kafkova S., Leonhardt K., Kellner C., Einenkel J. (2013). Serous tubal in situ carcinoma (STIC) in primary peritoneal serous carcinomas. Int. J. Gynecol. Pathol..

[26] Mehra K.K., Chang M.C., Folkins A.K., Raho C.J., Lima J.F., Yuan L., Mehrad M., Tworoger S.S., Crum C.P., Saleemuddin A. (2011). The impact of tissue block sampling on the detection of p53 signatures in fallopian tubes from women with BRCA 1 or 2 mutations (BRCA+) and controls. Mod. Pathol..

[27] Morrison J.C., Blanco L.Z., Vang R.V., Ronnett B.M. (2015). Incidental Serous Tubal Intraepithelial Carcinoma and Early Invasive Serous Carcinoma in the Nonprophylactic Setting Analysis of a Case Series. Am. J. Surg. Pathol..

[28] Meserve E.E., Mirkovic J., Conner J.R., Yang E., Muto M.G., Horowitz N., Stickland K.C., Howitt B.E., Crum C.P. (2017). Frequency of “incidental” serous tubal intraepithelial carcinoma (STIC) in women without a history of or genetic risk factor for high-grade serous carcinoma: A six-year study. Gynecol. Oncol..

[29] Rabban J.T., Garg K., Crawford B., Chen L., Zaouudek C.J. (2014). Early Detection of High-grade Tubal Serous Carcinoma in Women at Low Risk for Hereditary Breast and Ovarian Cancer Syndrome by Systematic Examination of Fallopian Tubes Incidentally Removed During Benign Surgery. Am. J. Surg. Pathol..

[30] Shaw P.A., Rouzbahaman M., Pizer E.S., Pintilie M., Begley H. (2009). Candidate serous cancer precursors in fallopian tube epithelium of BRCA1/2 mutation carriers. Mod. Pathol..

[31] Trabert B., Coburn S.B., Mariani A., Yang H.P., Rosenberg P.S., Gierach G.L., Wentzensen N., Cronin K.A., Sherman M.E. (2018). Reported incidence and survival of Fallopian tube carcinomas: A population-based analysis from the North American Association of Central Cancer Registries. J. Natl. Cancer Inst..

[32] Chen F., Gaitskell K., Garcia M.J., Albukhari A., Tsaltas J., Ahmed A.A. (2017). Serous tubal intraepithelial carcinomas associated with high-grade serous ovarian carcinomas: A systematic review. BJOG.

[33] Eckert M.A., Pan S., Hernandez K.M., Loth R.M., Andrade J., Volchenboum S.L., Faber P., Montag A., Lastra R., Peter M.E. (2016). Genomics of ovarian cancer progression reveals diverse metastatic trajectories including intraepithelial metastasis to the fallopian tube. Cancer Discov..

[34] Kim J., Coffey D.M., Ma L., Matzuk M.M. (2015). The ovary is an alternative site of origin for high-grade serous ovarian cancer in mice. Endocrinology.

[35] Hao D., Li J., Jia S., Meng Y., Zhang C., Wang L., Di L.J. (2017). Integrated analysis reveals tubal- and ovarian-originated serous ovarian cancer and predicts differential therapeutic responses. Clin. Cancer Res..

[36] Zhang S., Dolgalev I., Zhang T., Ran H., Levine D.A., Neel B.G. (2019). Both fallopian tube and ovarian surface epithelium are cells-of-origin for high-grade serous ovarian carcinoma. Nat. Commun..

[37] Coscia F., Watters K.M., Curtis M., Eckert M.A., Chiang C.Y., Tyanova S., Montag A., Lastra R.R., Lengyel E., Mann M. (2016). Integrative proteomic profiling of ovarian cancer cell lines reveals precursor cell associated proteins and functional status. Nat. Commun..

[38] Yates M.S., Meyer L.A., Deavers M.T., Daniels M.S., Keeler E.R., Mok S.C., Gershenson D.M., Lu K.H. (2011). Microscopic and early-stage ovarian cancers in BRCA1/2 mutation carriers: Building a model for early BRCA-associated tumorigenesis. Cancer Prev. Res..

[39] Soong T.R., Howitt B.E., Miron A., Horowitz N.S., Campbell F., Feltmate C.M., Muto M.G., Berkowitz R.S., Nucci M.R., Xian W. (2018). Evidence for lineage continuity between early serous proliferations (ESPs) in the Fallopian tube and disseminated high-grade serous carcinomas. J. Pathol..

[40] Terry K.L., Schock H., Fortner R.T., Husing A., Fichorova R.N., Yamamoto H.S., Vitonis A.F., Johnson T., Overvad K., Tjonneland A. (2016). A Prospective Evaluation of Early Detection Biomarkers for Ovarian Cancer in the European EPIC Cohort. Clin. Cancer Res..

[41] Hori S.S., Gambhir S.S. (2011). Mathematical model identifies blood biomarker-based early cancer detection strategies and limitations. Sci. Transl. Med..

[42] Zhang Q., Hu G., Yang Q., Dong R., Xie X., Ma D., Shen K., Kong B. (2013). A multiplex methylation-specific PCR assay for the detection of early-stage ovarian cancer using cell-free DNA. Gynecol. Oncol..

[43] Widschwendter M., Zikan M., Wahl B., Lempiainen H., Paprotka T., Evans I., Jones A., Ghazali S., Reisel D., Eichner J. (2017). The potential of circulating tumor DNA methylation analysis for the early detection and management of ovarian cancer. Genome Med..

[44] Cohen J.D., Li L., Wang Y., Thoburn C., Afsari B., Danilova L., Douville C., Javed A.A., Wong F., Mattox A. (2018). Detection and localization of surgically resectable cancers with a multi-analyte blood test. Science.

[45] Hayashi M., Matsuo K., Tanabe K., Ikeda M., Miyazawa M., Yasaka M., Machida H., Shida M., Imanishi T., Grubbs B.H. (2019). Comprehensive Serum Glycopeptide Spectra Analysis (CSGSA): A Potential New Tool for Early Detection of Ovarian Cancer. Cancers.

[46] Yang W.L., Gentry-Maharaj A., Simmons A., Ryan A., Fourkala E.O., Lu Z., Baggerly K.A., Zhao Y., Lu K.H., Bowtell D. (2017). Elevation of TP53 Autoantibody Before CA125 in Preclinical Invasive Epithelial Ovarian Cancer. Clin. Cancer Res..

[47] Diaz L.A., Bardelli A. (2014). Liquid biopsies: Genotyping circulating tumor DNA. J. Clin. Oncol..

[48] Takashina T., Ito E., Kudo R. (1985). Cytologic diagnosis of primary tubal cancer. Acta Cytol..

[49] Otsuka I., Kameda S., Hoshi K. (2013). Early detection of ovarian and fallopian tube cancer by examination of cytological samples from the endometrial cavity. Br. J. Cancer.

[50] Narutomi K., Umeki Y., Suwa S., Yamazaki N., Saito K., Fukase M., Saito N. (2001). A case of fallopian tube carcinoma in situ which was found by cervical and endometrial cytology. Tsuruoka Munic. Shonai Hosp. Med. J..

[51] Safret A., Bösch B., Bannwart F., Rinderknecht B., Hafner H.U. (2004). Carcinoma in situ of the fallopian tube presenting as a positive Pap smear. Acta Cytol..

[52] Doi D., Kamoi S., Tamura D., Kohana T., Iwata M. (1991). A case of primary fallopian tube carcinoma detected by mass screening. Saitama J. Obstet. Gynecol..

[53] Maeda D., Takazawa Y., Ota S., Takeuchi Y., Seta A., Nakagawa S., Yano T., Taketani Y., Fukayama M. (2010). Bilateral microscopic adenocarcinoma of the fallopian tubes detected by an endometrial cytologic smear. In. J. Gynecol. Pathol..

[54] Yamakawa Y., Teshima H., Hirai Y., Koi S., Fujimoto I., Yamauchi K., Hasumi K., Masubuchi K., Tsuzuki M. (1991). A case report of microinvasive carcinoma of the fallopian tube detected by endometrial cytology. J. Jpn. Soc. Clin. Cytol..

[55] Iida K., Takahashi Y., Shozu M., Kawakami K., Takatsuka F., Hata K., Yanagihara Y. (1989). A case of primary fallopian tube cancer detected by mass screening. Sanfujinka no Jissai.

[56] Konishi M., Mori E., Noguchi Y., Yamaguchi M., Bou Y., Tateyama N., Nishida N., Fukami T., Matsushima T., Doi D. (2011). A case with microscopic invasive primary carcinoma of fallopian tube that was detected by endometrial cytology. Kanagawa J. Obstet. Gynecol..

[57] Warshal D.P., Burgelson E.R., Aikins J.K., Rocereto T.F. (1999). Post-hysterectomy fallopian tube carcinoma presenting with a papanicolaou smear. Obstet. Gynecol..

[58] Kawanishi Y., Ito K., Uchida A., Hada K., Hayakasi Y., Kobayashi N., Hirayama E., Oikawa M., Okuyama K., Hareyama H. (2009). A case of fallopian tube carcinoma detected by positive endometrial cytology. Hokkaido Bull. Jpn. Soc. Clin. Cytol..

[59] Ikarashi H., Kodama S., Yoshiya N., Tanaka K., Nagai E., Emura I., Watanabe T., Sugai M. (1995). Case report of carcinoma in situ of the fallopian tube found by mass screening for uterine cancer. J. Jpn. Soc. Clin. Cytol..

[60] Minato H., Shimizu M., Hirokawa M., Fujiwara K., Kohno I., Manabe T. (1998). Adenocarcinoma in situ of the fallopian tube. A case report. Acta Cytol..

[61] Fujimoto T., Kiuchi C., Yuasa M., Tani K., Honda S., Yamao N., Miyashita H., Yamaguchi I. (1989). A case of carcinoma in situ of the fallopian tube. Kobe Chuo Hosp. Med. J..

[62] Imamura H., Hagiwara T., Kaneki E., Yahata H., Ogawa S., Kobayashi H., Kaku T. (2012). Endometrioid adenocarcinoma of the falloian tube, initially presenting as abnormal uterine cervical and endometrial cytology-A case report. J. Jpn. Soc. Clin. Cytol..

[63] Iwamoto H., Honda T., Fukasawa H., Nara M., Hashi A., Hirata S., Hoshi K. (2002). Primary cancer of the fallopian tube: The role of preoperative examinations. Nihon Fujinka Shuyo-Gakkai Zasshi.

[64] Takeda O., Takeda T., Sago H., Ochiai K., Onda T., Kitagawa M., Komuro N., Yasuda M., Sugishita T., Terashima Y. (1991). A case of occult ovarian carcinoma detected by endometrial cytologic examination. Tokyo Bull. Jpn. Soc. Clin. Cytol..

[65] Suzuki T., Bessho E., Tsuchiya K., Yamashita K. (1985). A case of primary minute carcinoma of a fallopian tube detected by repeated endometrial cytologic examination. Kinki Chuo Hosp. Res. J..

[66] Luzzatto R., Sisson G., Luzzatto L., Recktenvald M., Brucker N. (1996). Psammoma bodies and cells from in situ fallopian tube carcinoma in endometrial smears. A case report. Acta Cytol..

[67] Miyao Y., Ohara S., Kunitomo T. (2011). Primary fallopian tube carcinoma detected by endometrial cytology. Okayama Bull. Jpn. Soc. Clin. Cytol..

[68] Ohta H., Otsuka I., Ueda K., Lin Y.H., Akimoto N., Sugibayashi R., Furusawa Y., Suzuki M., Koi H., Kusanishi H. (2009). A case of a mixed endometrioid and clear cell adenocarcinoma arising from the fimbria of the Fallopian tube. Kanto J. Obstet. Gynecol..

[69] Kinde I., Bettegowda C., Wang Y., Wu J., Agrawal N., Shih I.M., Kurman R., Dao F., Levine D.A., Giuntoli R. (2013). Evaluation of DNA from the Papanicolaou test to detect ovarian and endometrial cancers. Sci. Transl. Med..

[70] Erickson B.K., Kinde I., Dobbin Z.C., Wang Y., Martin J.Y., Alvarez R.D., Conner M.G., Huh W.K., Roden R.B.S., Kinzler K.W. (2014). Detection of somatic TP53 mutation in tampons of patients with high-grade serous ovarian cancer. Obstet. Gynecol..

[71] Wang Y., Li L., Douville C., Cohen J.D., Yen T.T., Kinde I., Sundfelt K., Kjor S.K., Hruban R.H., Shih I.-M. (2018). Evaluation of liquid from the Papanicolaou test and other liquid biopsies for the detection of endometrial and ovarian cancers. Sci. Transl. Med..

[72] Maritschnegg E., Wang Y., Pecha N., Horvat R., Van Nieuwenhuysen E., Vergote I., Heitz F., Sehouli J., Kinde I., Diaz L.A. (2015). Lavage of the uterine cavity for molecular detection of Müllerian duct carcinomas: A proof-of concept study. J. Clin. Oncol..

[73] Barnabas G.D., Bahar-Shany K., Sapoznik S., Helpman L., Kadan Y., Beiner M., Weitzner O., Arbib N., Korach J., Perri T. (2019). Microvesicle Proteomic Profiling of Uterine Liquid Biopsy for Ovarian Cancer Early Detection. Mol. Cell. Proteom..

[74] Lum D., Guido R., Rodriguez E., Lee T., Mansuria S., D’Ambrosio L., Austin R.M. (2014). Brush cytology of the fallopian tube and implications in ovarian cancer screening. J. Minim. Invasive Gynecol..

[75] Zhou J., Gong G., Tan H., Dai F., Zhu X., Chen Y., Wang J., Liu Y., Chen P., Wu X. (2015). Urinary microRNA-30a-5p is a potential biomarker for ovarian serous adenocarcinoma. Oncol. Rep..

[76] Amal H., Shi D.Y., Ionescu R., Zhang W., Hua Q.L., Pan Y.Y., Tao L., Liu H., Haick H. (2015). Assessment of ovarian cancer conditions from exhaled breath. Int. J. Cancer.

[77] Yokoi A., Yoshioka Y., Hirakawa A., Yamamoto Y., Ishikawa M., Ikeda S., Kato T., Niimi K., Kajiyama H., Kikkawa F. (2017). A combination of circulating miRNAs for the early detection of ovarian cancer. Oncotarget.

[78] Elias K.M., Fendler W., Stawiski K., Fiascone S.J., Vitonis A.F., Berkowitz R.S., Frendl G., Konstantinopoulos P., Crum C.P., Kedzierska M. (2017). Diagnostic potential for a serum miRNA neural network for detection of ovarian cancer. eLife.

[79] McAlpine J.N., Hallani S.E.l., Lam S.F., Kalloger S.E., Luk M., Huntsman D.G., MacAulay C., Gilks C.B., Miller D.M., Lane P.M. (2011). Autofluorescence imaging can identify preinvasive or clinically occult lesions in fallopian tube epithelium: A promising step towards screening and early detection. Gynecol. Oncol..

[80] Williams R.M., Lee C., Galassi T.V., Harvey J.D., Leicher R., Sirenko M., Dorso M.A., Shah J., Olvera N., Dao F. (2018). Noninvasive ovarian cancer biomarker detection via an optical nanosensor implant. Sci. Adv..

[81] Ferenczy A., Gelfand M.M. (1984). Outpatient endometrial sampling with Endocyte: Comparative study of its effectiveness with endometrial biopsy. Obstet. Gynecol..

[82] Antoniou A.C., Rookus M., Andrieu N., Brohet R., Chang-Claude J., Peock S., Cook M., Evans D.G., Eeles R., Nogues C. (2009). Reproductive and hormonal factors, and ovarian cancer risk for *BRCA1* and *BRCA2* mutation carriers: Results from the International *BRCA1/2* Carrier Cohort Study. Cancer Epidemiol. Biomark. Prev..

[83] Yang H.P., Trabert B., Murphy M.A., Sherman M.E., Sampson J.N., Brinton L.A., Hartge P., Hollenbeck A., Park Y., Wentzensen N. (2012). Ovarian cancer risk factors by histologic subtypes in the NIH-AARP Diet and Health Study. Int. J. Cancer.

[84] Fathalla M.F. (1971). Incessant ovulation—A factor in ovarian neoplasia?. Lancet.

[85] Salvador S., Gilks B., Köbel M., Huntsman D., Rosen B., Miller D. (2009). The fallopian tube: Primary site of most pelvic high-grade serous carcinomas. Int. J. Gynecol. Cancer.

[86] Bahar-Shany K., Brand H., Sapoznik S., Jacob-Hirsch J., Yung Y., Korach J., Perri T., Cohen Y., Houvitz A., Levanon K. (2014). Exposure of fallopian tube epithelium to follicular fluid mimics carcinogenic changes in precursor lesions of serous papillary carcinoma. Gynecol. Oncol..

[87] Sieh W., Salvador S., McGuire V., Weber R.P., Terry K.L., Rossing M.A., Risch H., Wu A.H., Webb P.M., Moysich K. (2013). Tubal ligation and risk of ovarian cancer subtypes: A pooled analysis of case-control studies. Int. J. Epidemiol..

[88] Vercellini P., Crosignani P., Somigliana E., Vigano P., Buggio L., Bolis G., Fedele L. (2011). The ‘incessant menstruation’ hypothesis: A mechanistic ovarian cancer model with implications for prevention. Hum. Reprod..

[89] Shigeta S., Toyoshima M., Kitatani K., Ishibashi M., Usui T., Yaegashi N. (2016). Transferrin facilitates the formation of DNA double-strand breaks via transferrin receptor 1: The possible involvement of transferrin in carcinogenesis of high-grade serous ovarian cancer. Oncogene.

[90] Riman T., Dickman P.W., Nilsson S., Correia N., Nordlinder H., Magnusson C.M., Weiderpass E., Persson I.R. (2002). Hormone replacement therapy and the risk of invasive epithelial ovarian cancer in Swedish women. J. Natl. Cancer Inst..

[91] Koskela-Niska V., Riska A., Lyytinen H., Pukkala E., Ylikorkala O. (2012). Primary fallopian tube carcinoma risk in users of postmenopausal hormone therapy in Finland. Gynecol. Oncol..

[92] Koskela-Niska V., Pukkala E., Lyytinen H., Ylikorkala O., Dyba T. (2013). Effect of various forms of postmenopausal hormone therapy on the risk of ovarian cancer—A population-based case control study from Finland. Int. J. Cancer.

[93] Simin J., Tamimi R., Lagergren J., Adami H.-O., Brusselaers N. (2017). Menopausal hormone therapy and cancer risk: An overestimated risk?. Eur. J. Cancer.

[94] Kaunitz A.W., Manson J.E. (2015). Management of Menopausal Symptoms. Obstet. Gynecol..

[95] Luciano A.A., De Souza M.J., Roy M.P., Schoenfeld M.J., Nulsen J.C., Halvorson C.V. (1993). Evaluation of low-dose estrogen and progestin therapy in postmenopausal women. A double-blind, prospective study of sequential versus continuous therapy. J. Reprod. Med..

[96] Vang R., Shih I.M., Kurman R.J. (2013). Fallopian tube precursors of ovarian low- and high-grade serous neoplasms. Histopathology.

[97] George S.H., Milea A., Shaw P.A. (2012). Proliferation in the normal FTE is a hallmark of the follicular phase, not BRCA mutation status. Clin. Cancer Res..

[98] Kauff N.D., Domcheck S.M., Friebel T.M., Robson M.E., Lee J., Garber J.E., Isaacs C., Evans D.G., Lynch H., Eeles R.A. (2008). Risk-reducing salpingo-oophorectomy for the prevention of BRCA1- and BRCA2-associated breast and gynecologic cancer: A multicenter, prospective study. J. Clin. Oncol..

[99] Kotsopoulos J., Gronwald J., Karlan B., Rosen B., Huzarski T., Moller P., Lynch H.T., Singer C.F., Senter L., Neuhausen S.L. (2018). Age-specific ovarian cancer risks among women with a BRCA1 or BRCA2 mutation. Gynecol. Oncol..

[100] Nebgen D.R., Hurteau J., Holman L.L., Bradford A., Munsell M.F., Soletsky B.R., Sun C.C., Chisholm G.B., Lu K.H. (2018). Bilateral salpingectomy with delayed oophorectomy for ovarian cancer risk reduction: A pilot study in women with BRCA1/2 mutations. Gynecol. Oncol..

[101] Gan C., Chenoy R., Chandrasekaran D., Brockbank E., Hollingworth A., Vimplis S., Lawrence A.C., Jeyarajah A.R., Oram D., Deo N. (2017). Persistence of fimbrial tissue on the ovarian surface after salpingectomy. Am. J. Obstet. Gynecol..

[102] Gockley A.A., Elias K.M. (2018). Fallopian tube tumorigenesis and clinical implications for ovarian cancer risk-reduction. Cancer Treat. Rev..

[103] Mavaddat N., Peock S., Frost D., Ellis S., Platte R., Fineberg E., Evans D.G., Izatt L., Eeles R.A., Adlard J. (2013). Cancer risks for BRCA1 and BRCA2 mutation carriers: Results from prospective analysis of EMBRACE. J. Natl. Cancer Inst..

[104] Schrader K.A., Hurlburt J., Kalloger S.E., Hansford S., Young S., Huntsman D.G., Gilks B., McAlpine J.N. (2012). Germline BRCA1 and BRCA2 mutations in ovarian cancer: Utility of a histology-based referral strategy. Obstet. Gynecol..

[105] McLaughlin J.R., Risch H.A., Lubinski J., Moller P., Ghadirian P., Lynch H., Karlan B., Fishman D., Rosen B., Neuhausen S.L. (2007). Reproductive risk factors for ovarian cancer in carriers of *BRCA1* or *BRCA2* mutations: A case-control study. Lancet Oncol..

[106] Kotsopoulos J., Lubinski J., Gronwald J., Cybulski C., Demsky R., Neuhausen S.L., Kim-Sing C., Tung N., Friedman S., Senter L. (2015). Factors influencing ovulation and the risk of ovarian cancer in BRCA1 and BRCA2 mutation carriers. Int. J. Cancer.

[107] Benson L.S., Micks E.A. (2015). Why Stop Now? Extended and Continuous Regimens of Combined Hormonal Contraceptive Methods. Obstet. Gynecol. Clin. North Am..

[108] Trabert B., Ness R.B., Lo-Ciganic W.H., Murphy M.A., Goode E.L., Poole E.M., Brinton L.A., Webb P.M., Nagle C.M., Jordan S.J. (2014). Aspirin, nonaspirin nonsteroidal antiinflammatory drug, and acetaminophen use and risk of invasive epithelial ovarian cancer: A pooled analysis in the Ovarian Cancer Association Consortium. J. Natl. Cancer Inst..

[109] Del Pup L., Codacci-Pisanelli G., Peccatori F. (2019). Breast cancer risk of hormonal contraception: Counselling considering new evidence. Crit. Rev. Oncol. Hematol..

[110] Iversen L., Fielding S., Lidegaard O., Morch L.S., Skovlund C.W., Hannaford P.C. (2018). Association between contemporary hormonal contraception and ovarian cancer in women of reproductive age in Denmark: Prospective, nationwide cohort study. BMJ.

[111] Wu N.Y., Huang H.S., Chao T.H., Chou H.M., Fang C., Qin C.Z., Lin C.Y., Chu T.Y., Zhou H.H. (2017). Progesterone Prevents High-Grade Serous Ovarian Cancer by Inducing Necroptosis of p53-Defective Fallopian Tube Epithelial Cells. Cell Rep..

[112] Manson J.E., Aragaki A.K., Rossouw J.E., Anderson G.L., Prentice R.L., LaCroix A.Z., Chlebowski R.T., Howard B.V., Thomson C.A., Margolis K.L. (2017). Menopausal hormone therapy and long-term all-cause and cause-specific mortality. The Women’s Health Initiative Randomized Trials. JAMA.

[113] Elezaby M., Lees B., Maturen K.E., Barroilhet L., Wisinski K.B., Schrager S., Wilke L.G., Sadowski E. (2019). BRCA Mutation Carriers: Breast and Ovarian Cancer Screening Guidelines and Imaging Considerations. Radiology.

[114] Walsh T., Casadei S., Lee M.K., Pennil C.C., Nord A.S., Thornton A.M., Roeb W., Agnew K.L., Stray S.M., Wickramanayake A. (2011). Mutations in 12 genes for inherited ovarian, fallopian tube, and peritoneal carcinoma identified by massively parallel sequencing. Proc. Natl. Acad. Sci. USA.

[115] Norquist B.M., Harrell M.I., Brady M.F., Walsh T., Lee M.K., Gulsuner S., Bernards S.S., Casadei S., Yi Q., Burger R.A. (2016). Inherited Mutations in Women With Ovarian Carcinoma. JAMA Oncol..

[116] Kurian A.W., Ward K.C., Howlader N., Deapen D., Hamilton A.S., Mariotto A., Miller D., Penberthy L.S., Katz S.J. (2019). Genetic Testing and Results in a Population-Based Cohort of Breast Cancer Patients and Ovarian Cancer Patients. J. Clin. Oncol..

[117] Pal T., Permuth-Wey J., Betts J.A., Krischer J.P., Fiorica J., Arango H., LaPolla J., Hoffman M., Martino M.A., Wakeley K. (2005). *BRCA1* and *BRCA2* mutations account for a large proportion of ovarian carcinoma cases. Cancer.

[118] Zhang S., Royer R., Li S., McLaughlin J.R., Rosen B., Risch H.A., Fan I., Bradley L., Shaw P.A., Narod S.A. (2011). Frequencies of BRCA1 and BRCA2 mutations among 1,342 unselected patients with invasive ovarian cancer. Gynecol. Oncol..

[119] Enomoto T., Aoki D., Hattori K., Jinushi M., Kigawa J., Takeshima N., Tsuda H., Watanabe Y., Yoshihara K., Sugiyama T. (2019). The first Japanese nationwide multicenter study of BRCA mutation testing in ovarian cancer: CHARacterizing the cross-sectionaL approach to Ovarian cancer geneTic TEsting of BRCA (CHARLOTTE). Int. J. Gynecol. Cancer.

[120] Nielsen F.C., van Overeem Hansen T., Srensen C.S. (2016). Hereditary breast and ovarian cancer: New genes in confined pathways. Nat. Rev. Cancer.

[121] Visvanathan K., Shaw P., May B.J., Bahadirli-Talbott A., Kaushiva A., Risch H., Narod S., Wang T.L., Parkash V., Vang R. (2018). Fallopian Tube Lesions in Women at High Risk for Ovarian Cancer: A. Multicenter Study. Cancer Prev. Res..

[122] Pennington K.P., Walsh T., Harrel M.I., Lee M.K., Pennil C.C., Rendi M.H., Thomton A., Norquist B.M., Casadel S., Nord A.S. (2014). Germline and Somatic Mutations in Homologous Recombination Genes Predict Platinum Response and Survival in Ovarian, Fallopian Tube, and Peritoneal Carcinomas. Clin. Cancer Res..

[123] Labidi-Galy S.I., Papp E., Hallberg D., Niknafs N., Adleff V., Noe M., Bhattachaya R., Novak M., Jones S., Phallen J. (2017). High grade serous ovarian carcinomas originate in the fallopian tube. Nat. Commun..

[124] Wu R.C., Wang P., Lin S.F., Zhang M., Song Q., Chu T., Wang B.G., Kurman R.J., Vang R., Kinzler K. (2019). Genomic landscape and evolutionary trajectories of ovarian cancer precursor lesions. J. Pathol..

[125] Cabasag C.J., Arnold M., Butler J., Inoue M., Trabert B., Webb P.M., Bray F., Soerjomataram I. (2020). The influence of birth cohort and calendar period on global trends in ovarian cancer incidence. Int. J. Cancer.

[126] Yang H.P., Anderson W.F., Rosenberg P.S., Trabert B., Gierach G.L., Wentzensen N., Cronin K.A., Sherman M.E. (2013). Ovarian cancer incidence trends in relation to changing patterns of menopausal hormone therapy use in the united states. J. Clin. Oncol..

[127] Murakami R., Matsumura N., Michimae H., Tanabe H., Yunokawa M., Iwase H., Sasagawa M., Nakamura T., Tokuyama O., Takano M. (2019). The mesenchymal transition subtype more responsive to dose dense taxane chemotherapy combined with carboplatin than to conventional taxane and carboplatin chemotherapy in high grade serous ovarian carcinoma: A survey of Japanese Gynecologic Oncology Group study (JGOG3016A1). Gynecol. Oncol..

